# Cell Ratio Differences in Peripheral Blood between Early- and Late-Onset Parkinson's Disease: A Case-Control Study

**DOI:** 10.1155/2019/2072635

**Published:** 2019-11-07

**Authors:** Sen Jiang, Yuling Wang, Hua Gao, Qin Luo, Dan Wang, Yanxia Li, Yuxuan Yong, Xinling Yang

**Affiliations:** ^1^Department of Neurology, Second Affiliated Hospital of Xinjiang Medical University, No. 38, Nanhudonglu Road, Urumqi 830054, China; ^2^Medicine VIP, First Affiliated Hospital of Xinjiang Medical University, No. 137, Liyushanlu Road, Urumqi 830011, China; ^3^Department of Neurology, Fifth Affiliated Hospital of Xinjiang Medical University, No. 118, Henanxilu Road, Urumqi 830000, China; ^4^Department of Medicine, Tumor Hospital Affiliated of Xinjiang Medical University, No. 789, Suzhoudongjie Road, Urumqi 830000, China; ^5^Department of Rehabilitation, Second Affiliated Hospital of Xinjiang Medical University, No. 38, Nanhudonglu Road, Urumqi 830054, China

## Abstract

**Objectives:**

To explore the differences of immune disorders in peripheral blood between patients with early-onset Parkinson's disease (EOPD) and late-onset Parkinson's disease (LOPD).

**Methods:**

We retrospectively reviewed medical records of Parkinson's disease (PD) patients and healthy controls between June 2002 and July 2017. At last, we included 117 PD patients who were divided into EOPD and LOPD according to whether onset age of PD was after 50 and 99 controls divided into E-Control (match for EOPD) and L-Control (match for LOPD) according to whether their age was after 53 which was onset age plus median of disease duration. We compared the ratios of cells between multiple groups and performed the multinominal logistic regression analysis to explore the relationship between ratios and subtypes of PD. We also carried out the receiver operating characteristic (ROC) curve analysis to estimate the diagnostic value of the variable.

**Results:**

Lymphocyte-red blood cell ratio (LRR) was lower in LOPD compared with that in EOPD or L-Control. LRR was also negatively associated with LOPD (OR: 0.623; 95% CI: 0.397–0.980; *P*=0.040). The ROC curve analysis showed the optimal cutoff value of 4.53 (×10^−4^) of LRR for discrimination of LOPD versus L-Control (sensitivity: 0.596, specificity: 0.764). The area under curve (AUC) was 0.721. As for LOPD versus EOPD, the optimal threshold of LRR was 4.10 (×10^−4^) (sensitivity: 0.516, specificity: 0.745). AUC was 0.641.

**Conclusions:**

Peripheral immune disorders might play an important part in the pathological progression of LOPD. Also, LRR has potential diagnostic value.

## 1. Introduction

Parkinson's disease (PD) is a neurodegenerative disorder with the pathological change of degeneration of substantia nigra and clinical characteristics of bradykinesia, rigidity, and resting tremor. The onset age of PD is usually after 50. However, it is often found in clinical practice that some patients show symptoms of PD at a young age. These patients have relatively unique clinical features, such as tremor with small amplitude and fast frequency with relatively good response to drug treatment, yet rarely pill-rolling tremor [1]. Therefore, researchers could classify PD into early-onset PD (EOPD) and late-onset PD (LOPD) based on the patient's onset age.

However, there is some controversy regarding the onset age for discriminate EOPD versus LOPD. Not all researchers accept age 50, and some still define the age 40 or 45 as the dividing line. However, a study with large sample of twins in 1999 showed that genetic factors play an important role in the pathological progression of PD with onset before age 50 but have no effects on that with onset after age 50 [2]. Then, subsequent research suggested that effects of genetic factors on LOPD are smaller than those on EOPD [3]. Therefore, it is reasonable to define age of 50 years as the dividing line between EOPD and LOPD based on the effects of genetic factors.

Recently, some studies showed the evidence for the role of immune disorders in the pathological progression of PD, which gets more and more attention. For example, microglia in Central Nervous System (CNS) gets activated [4] and releases the inflammatory factors [5]; lymphocyte regulates the inflammatory response in CNS [6]; some peripheral immune cells change their numbers during the progression of PD [7]; and neutrophil-lymphocyte ratio (NLR) decreases in PD patients [8]. However, no studies have ever reported the relationship between immune factor and EOPD or LOPD.

Peripheral blood cells that involve neutrophil (NEU), lymphocyte (LY), monocyte (MO), eosinophil (EO), basophil (BA), red blood cell (RBC), and platelet (PLT) have certain immune capabilities. Based on the counts of the cells, we can calculate some ratios that reflect information from two different cells at the same time to indicate the disorders of peripheral immune function, such as NLR, which usually plays a prognostic role in cancer [9]. In this study, we compared the ratios in EOPD patients with those in LOPD patients and used the multinominal logistic regression analysis to explore the association of peripheral immune disorders with EOPD and LOPD. We also performed receiver operating characteristic (ROC) curve analysis to estimate the potential diagnostic value of significant indicator.

## 2. Methods

### 2.1. Participants

We retrospectively reviewed the medical records of subjects who sought for health checkup or treatment for PD in First Affiliated Hospital of Xinjiang Medical University between June 2002 and July 2017. Their medical records included the patients' information, such as age, gender, duration of disease, smoking history, symptoms and signs at admission, results of auxiliary examination, and diagnosis at discharge. The severity of PD was quantified by the modified Hoehn–Yahr (HY) Scale.

Two neurologists asked patients who sought for treatment for PD about their history of present illness and past history, performed the physical examination on them, and decided which auxiliary examinations are needed, such as Blood Routine (BR) Examination and Magnetic Resonance Imaging. At last, the two neurologists made the diagnosis following the United Kingdom Parkinson's Disease Society Brain Bank Clinical Diagnostic Criteria [10].

PD group inclusive criteria were diagnosis of PD, normal nutritional status, and no diarrhea.

Control group inclusive criteria were healthy individuals matched to PD patients on age, gender, and smoking history; normal nutritional status; and no diarrhea.

Exclusive criteria were as follows: be vaccinated within 3 months; use of immunosuppressant or immune booster within 3 months; chronic neurodegenerative disease other than PD; cancer; infectious disease; chronic inflammation; and systemic disease.

At last, we did not get enough controls to apply 1 : 1 match and included a total of 117 PD patients and 99 controls into this study. The dividing line for onset age was age 50 [2]. Of the 117 PD patients, 62 were EOPD and 55 were LOPD. The median of disease duration of all patients was 3 years. So we divided the controls according to age of 53 years which was onset age plus disease duration. Of 99 controls, 42 were matched to EOPD patients (E-Control) and 57 were matched to LOPD patients (L-Control).

When the participants were admitted to the hospital, they signed an informed consent to declare to agree on sharing their medical information for research. The study was carried out in accordance with the Helsinki Declaration. The protocol was approved by the Ethics Committee of the First Affiliated Hospital of Xinjiang Medical University.

### 2.2. Ratios of Cells

The nurse took the blood sample from the vein in the antecubital fossa of patients with an empty stomach on the second day after admission. An automated hematology analyzer (SYSMEX 2000; Sysmex Corp., Kobe, Japan) analyzed the blood sample. The result provided the counts of 7 kinds of cells, NEU, LY, MO, EO, BA, RBC, and PLT. We can calculate the different ratios by dividing any two numbers of cells, including NLR, neutrophil-monocyte ratio (NMR), basophil-neutrophil ratio (BNR), eosinophil-neutrophil ratio (ENR), lymphocyte-monocyte ratio (LMR), eosinophil-lymphocyte ratio (ELR), basophil-lymphocyte ratio (BLR), eosinophil-monocyte ratio (EMR), basophil-monocyte ratio (BMR), basophil-eosinophil ratio (BER), neutrophil-platelet ratio (NPR), lymphocyte-platelet ratio (LPR), monocyte-platelet ratio (MPR), eosinophil-platelet ratio (EPR), basophil-platelet ratio (BPR), neutrophil-red blood cell ratio (NRR), lymphocyte-red blood cell ratio (LRR), monocyte-red blood cell ratio (MRR), eosinophil-red blood cell ratio (ERR), basophil-red blood cell ratio (BRR), and platelet-red blood cell ratio (PRR).

### 2.3. Statistical Analysis

We used SPSS 22.0 software (SPSS Inc., Chicago, IL, USA) for statistical analysis and entered all data into the software. The qualitative variables were assessed by Pearson's Chi-squared test or Fisher's exact test. The normality of quantitative data was detected by analytic methods (e.g., Kolmogorov–Smirnov test or Shapiro–Wilk test). When the variable was compliant with normal distribution, it was presented as mean (standard deviations (SD)). We used one way analysis of variance (one-way ANOVA) to compare means between multiple groups. When the variable was not normally distributed, it was presented as median (interquartile (IQR)). We used the Mann–Whitney *U* test for comparison between two groups and the Kruskal–Wallis test between multiple groups.

In addition, we used the multinominal logistic regression analysis of significant variables with EOPD or LOPD to explore the association of ratios of cells with subtypes of PD. Then we performed ROC curve analysis to estimate the diagnostic value of the variable. The value with the largest Youden index was the optimal cutoff point. We also calculated area under curve (AUC).

If the missing data was less than 5% of the sample, we would not carry out any process. All tests were 2-sided. *P* < 0.05 was statistically significant.

## 3. Results

### 3.1. Comparison of Characteristics between Groups

Table 1 shows the comparisons of baseline characteristics. EOPD group included 62 patients; LOPD included 55; E-Control included 42; and L-Control included 57. The median onset age in EOPD was significantly lower than that in LOPD. There were differences of age between any two groups except for EOPD versus E-Control or LOPD versus L-Control (*P* > 0.999). Also, there were no differences of duration of PD and HY scales between EOPD and LOPD and no smoking history and gender differences between any two groups.

### 3.2. Comparison of Ratios between Groups

We compared different ratios between multiple groups (Table 2). We excluded 2 patients because of their incomplete data, which was the error occurred randomly in the transition from paper medical records to electronic medical records. There were increment of NLR and decrement of LMR, LPR, and LRR in LOPD relative to L-Control (*P*=0.003, *P*=0.017, *P*=0.018, and *P* < 0.001). LRR also decreased in LOPD relative to EOPD or E-Control (*P*=0.018, *P*=0.003). As for E-Control versus L-Control, only BER was higher in E-Control (*P*=0.014). When we compared EOPD with L-Control, LPR and LRR decreased in EOPD (*P*=0.004, *P*=0.037), while BER increased (*P*=0.024).

### 3.3. Multinominal Logistic Regression Analysis of Significant Variables

We included 61 EOPD patients, 54 LOPD patients, 42 E-Controls, and 57 L-Controls in the model. The group was dependent variable (EOPD = 1, LOPD = 2, E-Control = 3, and L-Control = 4). We also included age, sex, and smoking history to adjust the model and NLR, LMR, BER, LPR, and LRR as independent variables, the variance inflation factors of which were all smaller than 2 (data not shown), indicating no existence of multi-collinearity. The reference category was L-Control. The results are shown in Table 3. LRR was negatively associated with LOPD.

### 3.4. ROC Curve Analysis of LRR

**Figure 1:**
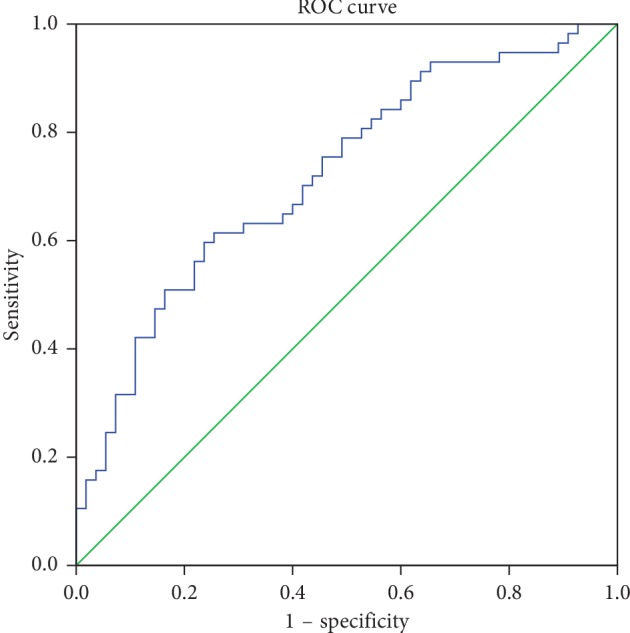
ROC curve for discrimination of LOPD versus L-Control.

**Figure 2:**
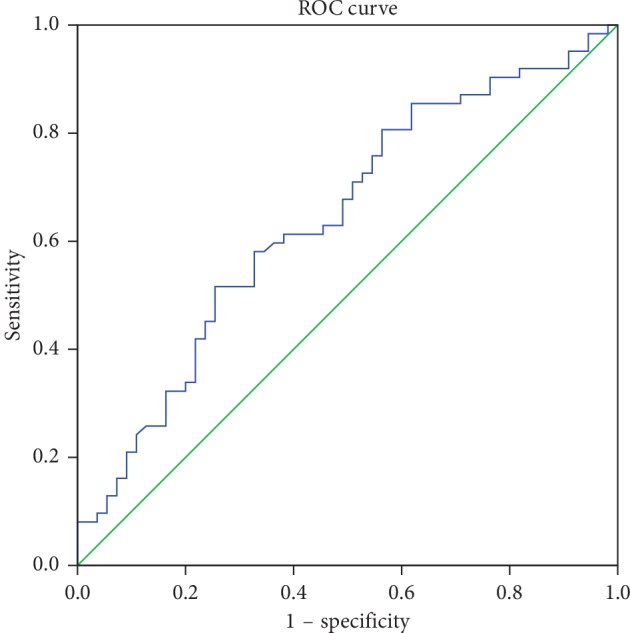
ROC curve for discrimination of LOPD versus EOPD.

We performed ROC curve analysis to estimate the diagnostic value of LRR (Figures 1 and 2). The data of 61 EOPD patients, 54 LOPD patients, and 57 L-Controls were available. The optimal cutoff value for discriminate LOPD versus L-Control was 4.53 (×10^−4^) when the largest Youden index was 0.360 (sensitivity: 0.596, specificity: 0.764). And, the AUC was 0.721 (Table 4). We also obtained an optimal cutoff value 4.10 (×10^−4^) with the largest youden index of 0.262 to differentiate LOPD versus EOPD (sensitivity: 0.516; specificity: 0.745). And, the AUC was 0.641 (Table 4).

## 4. Discussion

We found the decrement of LRR in LOPD compared with that in EOPD or L-Control. Besides, between LOPD and L-Control, we also found the increment of NLR and decrement of LMR and LPR in LOPD. After multinominal logistic regression analysis, we observed the association of LRR with LOPD. The ROC curve analysis also showed the LRR has diagnostic value for discriminate LOPD versus L-Control or LOPD versus EOPD. According to our search in the database, it is the first study to explore the differences of peripheral immune disorders between EOPD and LOPD patients.

The previous researches regarding EOPD and LOPD focused on the genetic factor, which has more effects on the pathological progression of EOPD [3], whereas, in our research, we studied the immune factor which is associated with LOPD. Some studies also have reported the relationship between ratios of blood cells and PD. As the commonly used indicator for systemic inflammation, some studies suggested the increment of NLR in PD patients. In addition, researchers found the NLR is not only higher in PD patients [8], but also positively associated with severity of PD [11]. Nevertheless, not all results are consistent. Someone compared NLR between patients with different clinical manifestations and observed no differences [12]. In this study, it was found that NLR was higher in LOPD patients compared with that in L-Control. However, based on our findings, LRR might play an more important role than the NLR in the pathological progression of LOPD.

There have been some evidence suggested that LY and RBC take their effects on the pathological progression of PD, such as the accumulation of T lymphocytes in CNS in animal model [13] and PD patients [14]. Whether lymphocyte activates the microglia or microglia leads to the accumulation of T lymphocytes in CNS is still not clear. However, the procession plays an important role in the inflammatory response in the CNS of PD patients. After being activated, microglia will release some inflammatory factors [5] to regulate the inflammatory response. On the other hand, the cellular immune response leads to death of dopamine (DA) neurons via CD4+ T lymphocytes-dependent Fas/Fasl [6]. Besides, someone also observed the decrement of CD3+ T lymphocytes [7] in peripheral blood of PD patients. It is still not clear how inflammatory response in CNS affects the peripheral immune cell. Because of lack of evidence, it is only an assumption that neuron-immune network has an effect on the progression.

One of the characteristic pathological manifestations of PD is the accumulation of Lewy bodies in neurons of CNS, which is composed of alpha-synuclein (*α*-syn) with rich content in CNS [15]. The *α*-syn oligomer in cerebrospinal fluid with more toxicity than *α*-syn monomer [16] can be used as the indicator for the diagnosis of Parkinson's disease [17]. In addition, *α*-syn can penetrate the blood-brain barrier to the peripheral blood. 99% of *α*-syn entered the blood accumulate in the RBC, and 0.1% are present in plasma [18]. High concentration of free iron in RBC might be the reason of aggregation of *α*-syn oligomers in these cells [19]. Furthermore, there is a paper showing the relationship between homocysteine (HCY) and PD [20], which reflects the oxidative stress in CNS. The metabolism of HCY requires vitamin B12 as coenzyme. Some studies also found the decrement of vitamin B12 in PD patients [21] that can lead to the megaloblastic anemia. Through above two routes, RBC plays an important part in the peripheral immune disorders of PD.

Moreover, it has been reported that RBC activates the immune function of T lymphocytes by interaction between CD58, CD59 on the surface of RBC, and CD2 on the surface of T helper cell [22]. RBC can also promote proliferation and differentiation of B lymphocytes to produce immunoglobulins [22]. Variation of LRR might reflect the information of the interaction between lymphocyte and RBC. Thus, we could make an assumption that interaction also has its effect on the pathological progression of LOPD.

This study is limited. First, doctors have recorded the accurate drug history in patients' medical records since only a few years ago. Unfortunately, drug history is not detailed enough before that. Because of the lack of these data, we cannot exclude confounding factors that the drugs individuals used in this study. Second, this is a hospital-based study. The selection of study subjects is not random. Thus, biases are certainly existent. Last, genetic factors have been proved to be related with EOPD [3]. However, because our research is a retrospective study, we cannot obtain the subjects' genetic information to analyze the interaction between immune and gene in PD patients.

In summary, not only do genetic factors differ between EOPD and LOPD, but also immune factors differ between these two kinds of PDs. LRR which showed potential diagnostic value was associated with LOPD, suggesting that there might be differences of inflammatory responses between EOPD and LOPD. The peripheral immune disorders are more serious in LOPD patients. However, the detailed mechanism is still unclear and only speculation could be made. More fundamental researches are needed.

## Figures and Tables

**Table 1:** Characteristics of the included population.

	EOPD	E-Control	LOPD	L-control	*P*
	(*n* = 62)	(*n* = 42)	(*n* = 55)	(*n* = 57)	
Onset age, median (IQR), *y*	44.00 (8.00)	—	57.00 (6.83)	—	<0.001a^*∗*^
Age, median (IQR), *y*	48.00 (7.00)	46.50 (5.00)	60.00 (6.00)	61.00 (9.00)	<0.001b^*∗*^
Duration, median (IQR), *y*	3.00 (3.50)	—	2.00 (3.00)	—	0.05^a^
HY, median (IQR)	2.00 (2.00)	—	2.00 (0.50)	—	0.602^a^
Sex					
Female (%)	29 (46.8)	26 (61.9)	29 (52.7)	34 (59.6)	0.376^c^
Male (%)	33 (53.2)	16 (38.1)	26 (47.3)	23 (40.4)	
Smoking					
No	52 (83.9)	37 (88.1)	46 (83.6)	45 (78.9)	0.684^c^
Yes	10 (16.1)	5 (11.9)	9 (16.4)	12 (21.1)	

^a^Mann–Whitney *U* test. ^b^Kruskal–Wallis test. ^c^Pearson's Chi-squared test. ^*∗*^*P* < 0.001.

**Table 2:** Intergroup comparison of cell ratios.

	EOPD	E-Control	LOPD	L-control	*P*
	(*n* = 61)	(*n* = 42)	(*n* = 54)	(*n* = 57)	
NLR, median (IQR)	1.733 (0.715)	1.888 (1.037)	2.039 (1.289)	1.539 (0.694)	0.008a^*∗*^
NMR, median (IQR)	8.194 (3.772)	8.663 (4.211)	9.370 (4.217)	9.027 (4.265)	0.275^a^
BNR, median (IQR), ×10^−2^	0.631 (0.717)	0.634 (0.764)	0.460 (0.459)	0.465 (0.723)	0.109^a^
ENR, median (IQR), ×10^−2^	3.543 (2.837)	3.322 (2.904)	3.301 (3.237)	3.721 (5.107)	0.315^a^
LMR, median (IQR),	5.042 (2.678)	5.283 (2.678)	4.274 (3.103)	5.405 (3.185)	0.011a^*∗*^
ELR, median (IQR), ×10^−2^	6.122 (4.300)	5.580 (5.153)	6.790 (6.975)	5.983 (6.442)	0.851^a^
BLR, median (IQR), ×10^−2^	1.020 (1.350)	1.105 (0.991)	0.833 (1.293)	0.823 (0.885)	0.066^a^
EMR, median (IQR),	0.290 (0.272)	0.309 (0.220)	0.288 (0.269)	0.388 (0.381)	0.139^a^
BMR, median (IQR), ×10^−2^	5.556 (7.438)	6.667 (5.978)	4.226 (4.298)	5.000 (6.388)	0.129^a^
BER, median (IQR),	0.188 (0.259)	0.200 (0.229)	0.155 (0.188)	0.125 (0.183)	0.035a^*∗*^
NPR, median (IQR), ×10^−2^	1.525 (0.675)	1.729 (1.046)	1.694 (0.990)	1.861 (0.999)	0.300^a^
LPR, median (IQR), ×10^−2^	0.872 (0.488)	0.873 (0.351)	0.854 (0.541)	1.077 (0.487)	0.003a^*∗*^
MPR, median (IQR), ×10^−2^	0.186 (0.089)	0.182 (0.087)	0.199 (0.137)	0.188 (0.112)	0.718^a^
EPR, median (IQR), ×10^−3^	0.519 (0.458)	0.582 (0.484)	0.510 (0.683)	0.700 (0.733)	0.214^a^
BPR, median (IQR), ×10^−3^	0.108 (0.131)	0.122 (0.112)	0.074 (0.085)	0.089 (0.104)	0.182^a^
NRR, median (IQR), ×10^−3^	0.758 (0.268)	0.861 (0.412)	0.798 (0.328)	0.726 (0.327)	0.703^a^
LRR, mean (SD), ×10^−4^	4.455 (1.166)	4.659 (1.335)	3.880 (1.048)	4.920 (1.404)	<0.001b^*∗*^
MRR, mean (SD), ×10^−4^	0.973 (0.291)	0.919 (0.349)	0.889 (0.302)	0.905 (0.322)	0.479^b^
ERR, median (IQR), ×10^−4^	0.270 (0.213)	0.252 (0.226)	0.261 (0.248)	0.291 (0.304)	0.344^a^
BRR, median (IQR), ×10^−4^	0.044 (0.067)	0.052 (0.057)	0.031 (0.048)	0.045 (0.053)	0.063^a^
PRR, median (IQR), ×10^−2^	4.784 (1.689)	4.942 (1.438)	4.538 (2.190)	4.518 (1.763)	0.205^a^

^a^Kruskal–Wallis test. ^b^one-way ANOVA. ^*∗*^*P* < 0.05.

**Table 3:** Multinominal logistic regression analysis of ratios of cells.

	*P*	OR	95% Confidence Interval for OR	
			Lower bound	Upper bound
EOPD				
Age	<0.001^*∗*^	0.548	0.455	0.661
NLR	0.567	0.748	0.277	2.019
LMR	0.280	0.863	0.660	1.128
BER	0.481	3.082	0.135	70.490
LPR	0.730	0.720	0.112	4.644
LRR	0.382	0.738	0.374	1.458
Sex				
Female	0.280	0.425	0.090	2.006
Smoking				
No	0.178	3.420	0.572	20.446
LOPD				
Age	0.348	0.964	0.892	1.041
NLR	0.328	1.383	0.723	2.644
LMR	0.480	1.008	0.986	1.030
BER	0.890	1.173	0.122	11.305
LPR	0.352	0.531	0.140	2.014
LRR	0.040^*∗*^	0.623	0.397	0.980
Sex				
Female	0.193	0.490	0.167	1.434
Smoking				
No	0.231	2.168	0.612	7.680
E-Control				
Age	<0.001^*∗*^	0.520	0.428	0.632
NLR	0.743	0.845	0.308	2.313
LMR	0.903	1.005	0.921	1.098
BER	0.588	2.411	0.100	58.033
LPR	0.680	0.650	0.084	5.020
LRR	0.455	0.764	0.377	1.549
Sex				
Female	0.765	0.778	0.149	4.057
Smoking				
No	0.203	3.639	0.497	26.648

^*∗*^ *P* < 0.05.

**Table 4:** ROC analysis of LRR.

	*P*	Area	95% confidence interval	
			Lower bound	Upper bound
L-control vs. LOPD	<0.001^*∗*^	0.721	0.627	0.814
EOPD vs. LOPD	0.008^*∗*^	0.641	0.541	0.742

^*∗*^ *P* < 0.05.

## Data Availability

The data that support the findings of this study are available on request from the corresponding author. The data are not publicly available due to privacy restrictions.
